# A Hand-Modeled Feature Extraction-Based Learning Network to Detect Grasps Using sEMG Signal

**DOI:** 10.3390/s22052007

**Published:** 2022-03-04

**Authors:** Mehmet Baygin, Prabal Datta Barua, Sengul Dogan, Turker Tuncer, Sefa Key, U. Rajendra Acharya, Kang Hao Cheong

**Affiliations:** 1 Department of Computer Engineering, College of Engineering, Ardahan University, Ardahan 75000, Turkey; mehmetbaygin@ardahan.edu.tr; 2 School of Business (Information System), University of Southern Queensland, Toowoomba, QLD 4350, Australia; prabal.barua@usq.edu.au; 3 Faculty of Engineering and Information Technology, University of Technology Sydney, Sydney, NSW 2007, Australia; 4 Cogninet Brain Team, Cogninet Australia, Sydney, NSW 2010, Australia; 5 Department of Digital Forensics Engineering, College of Technology, Firat University, Elazig 23119, Turkey; sdogan@firat.edu.tr (S.D.); turkertuncer@firat.edu.tr (T.T.); 6 Department of Orthopedics and Traumatology, Bingöl State Hospital, Ministry of Health, Bingöl 12000, Turkey; sefa_key@hotmail.com; 7 Department of Electronics and Computer Engineering, Ngee Ann Polytechnic, Singapore 599489, Singapore; 8 Department of Biomedical Engineering, School of Science and Technology, SUSS University, Singapore 599494, Singapore; 9 Department of Biomedical Informatics and Medical Engineering, Asia University, Taichung 41354, Taiwan; 10 Science, Mathematics and Technology Cluster, Singapore University of Technology and Design, Singapore 487372, Singapore

**Keywords:** frustum pattern, Frustum154, sEMG signal classification, grasp detection

## Abstract

Recently, deep models have been very popular because they achieve excellent performance with many classification problems. Deep networks have high computational complexities and require specific hardware. To overcome this problem (without decreasing classification ability), a hand-modeled feature selection method is proposed in this paper. A new shape-based local feature extractor is presented which uses the geometric shape of the frustum. By using a frustum pattern, textural features are generated. Moreover, statistical features have been extracted in this model. Textures and statistics features are fused, and a hybrid feature extraction phase is obtained; these features are low-level. To generate high level features, tunable Q factor wavelet transform (TQWT) is used. The presented hybrid feature generator creates 154 feature vectors; hence, it is named Frustum154. In the multilevel feature creation phase, this model can select the appropriate feature vectors automatically and create the final feature vector by merging the appropriate feature vectors. Iterative neighborhood component analysis (INCA) chooses the best feature vector, and shallow classifiers are then used. Frustum154 has been tested on three basic hand-movement sEMG datasets. Hand-movement sEMG datasets are commonly used in biomedical engineering, but there are some problems in this area. The presented models generally required one dataset to achieve high classification ability. In this work, three sEMG datasets have been used to test the performance of Frustum154. The presented model is self-organized and selects the most informative subbands and features automatically. It achieved 98.89%, 94.94%, and 95.30% classification accuracies using shallow classifiers, indicating that Frustum154 can improve classification accuracy.

## 1. Introduction

Electromyography (EMG) is a diagnostic procedure to assess the health of muscles and the nerve cells that control them (motor neurons). There are two types of EMG: intramuscular and surface [1,2,3]. Intramuscular EMG is recorded with the help of invasive electrodes. With surface EMG, on the other hand, noninvasive electrodes are used to detect the electrical signals of the muscle. Surface EMG is widely preferred to detect muscle activation time and density [4]. Electromyography (EMG) signals can be used to diagnose neuromuscular diseases through muscles and nerve cells that control muscles [5,6]. These nerve cells, known as motor neurons, transmit electrical signals that cause the muscle to contract and relax. These electrical signals may be recorded using different techniques, e.g., EMG signals obtained with the help of electrodes connected to surface, such as the hands and arms, or needles/wires connected to muscle tissue [7].

EMG signals are used in clinical applications to assist in the creation of devices such as prosthetic hands/arms [8,9,10]. Prosthetic hands/arms have been developed for amputees, disabled people, and patients with movement loss [11,12]. These devices can improve patient quality of life; however, they are also very costly. Current control systems and applied methodologies have improved over the years in terms of increasing the mobility of these devices. Many studies on the development of artificial intelligence-assisted myoelectric control-based smart devices have been presented [13]. Systems with a myoelectric interface can interact with devices and individuals. Thus, devices developed with systems involving myoelectric interfaces provide more efficient interactions [14]. The primary purpose of these devices is to provide patients with realistic and highly efficiency movement. In this respect, EMG signals must be interpreted and processed correctly. Many machine learning techniques have been developed for the automatic and effective processing of such signals. These methods are varied to ensure that the devices operate with high efficiency, and that the signal is interpreted correctly [15,16,17,18].

Many studies are present in the literature on the analysis of EMG signals to reduce expert dependence and minimize human error [19,20,21]. Menon et al. [22] developed a classification technique for forearm prosthetic devices. The technique uses EMG data describing seven hand gestures. The data were collected from 9 healthy individuals and 13 amputees. The study emphasized that the classification technique was of great importance for myoelectric prosthesis. The authors constructed five cases using EMG signals with lengths of 50 ms, 150 ms, 250 ms, 350 ms, and 450 ms. Moreover, they used a linear discriminant analysis (LDA) classifier, and their maximum classification accuracy was 95.44% using EMG signals with a length of 450 ms. Mukhopadhyaya and Samui [11] proposed a method to efficiently control prosthetic devices. The approach used the Deep Neural Network method to process the EMG signal. Khushaba [23] utilized a dataset containing eight hand gestures to test the performance of the method. In their model, they used an EMG dataset collected from five participants. A deep classifier (DNN (deep neural network)) yielded 98.88% classification accuracy. Waris et al. [24] evaluated the classification performance of EMG signals obtained from healthy individuals using upper limb prostheses. Seven-day data of the individuals was evaluated in the study. The EMG signal dataset was collected from eight transradial amputees and ten healthy participants. The authors used classifiers such as artificial neural network (ANN), tree, and LDA classifiers. Their presented machine learning model achieved over 90% accuracy using ANN. Chada et al. [25] proposed a method to provide robotic control using surface EMG signals. In their method, subbands of the signal were obtained by using Tunable-Q factor wavelet transform. The dataset was collected from five subjects. Various properties were obtained from each subband; these features were then classified using a radial basis function support vector machine (SVM), with an accuracy of 97.74%. Wang et al. [26] proposed an approach that could be used in rehabilitation devices for individuals with disabilities in their upper-limbs. They used a deep model to achieve high classification rates, reaching 92% accuracy using their recurrent deep model for six class classification. Their dataset contained EMG signals from 10 healthy subjects. Arteaga et al. [27] proposed an EMG signal-based approach for the modeling and analysis of hand movements of healthy individuals. The proposed approach used machine learning methods to evaluate EMG signals. In this study, six hand movements were selected, and data from 20 individuals were used. The classification results showed that the most successful results in the analysis of the data were obtained by k nearest neighbor (kNN). The accuracy rate was calculated as 98% for kNN. Pancholi and Joshi [28] presented a system for evaluating EMG signals which aims to recognize the structure of arm movements from EMG signals received from amputees. In the study, six different movements from four individuals were collected and evaluated. The highest accuracy rate was 97.75%, which was achieved using LDA classifier with hold-out validation. Jia et al. [29] proposed a method for classifying EMG signals using convolutional neural networks. The windowing method was used to improve the performance of the proposed method. In the utilized dataset, sEMG signals from eight participants were used; the accuracy rate was 99.38%. Tuncer et al. [4] proposed an approach to ensure proper hand movement with EMG signals, and data from nine transradial amputee patients were used. Discrete wavelet transform and ternary pattern were selected as feature extractors in theit study. The evaluation results were presented according to the following parameters: accuracy (99.14%), geometric mean (99.13%), precision (99.14%), and F1-score (99.14%), employing kNN classifier with 10-fold cross-validation. Simãoa et al. [30] developed a model for the classification of EMG signals obtained from forearm muscles. In the model, feature extraction was provided by recurrent neural networks. In addition, the study was compared with long short-term memory networks and gated recurrent unit methods. Time and accuracy rates were presented using DualMyo [31] and NinaPro DB5 [32] datasets. Their model achieved about 95% accuracy using DualMyo datasets [31], and 91% using NinaPro DB5 [32] EMG datasets.

sEMG signal classification is an important research topic for machine learning and biomedical engineering. In this work, we propose a hand-modeled learning method for sEMG signal classification with high performance. To achieve our goal, three sEMG datasets were used for testing. A new effective learning method was also applied to achieve high classification ability with linear time complexity with sEMG signals; this learning model was named Frustum154. The main aim of the Frustum154 model is to select the most valuable subbands to generate features. Frustum154 comprises three main phases: (i) feature extraction using the presented frustum pattern, statistical features, and multiple parameter-based, tunable Q actor wavelet transform (TQWT) [33] decomposition, (ii) iterative neighborhood component analysis (INCA) [34] selector, and (iii) classification using a support vector machine (SVM) [35,36] or kNN [37]. Frustum154 allows us to propose a systematic hand-crafted method. It can also choose the most appropriate model for signal classification problems.

The key novelties and contributions of this model are given below:

Novelties:

- Shapes can be used to propose new local textural feature generators. Therefore, the frustum shape is used to present a new textural feature creation function, named the “frustum pattern”. By using the frustum pattern, a shape-related, graph-based local feature extraction methodology is investigated in this work.
- A new learning network called Frustum154 is presented in this paper. Frustum154 is a self-organized learning feature extraction method which uses two types of feature selection. In the feature generation/creation phase, the best features are chosen using a loss function. By using this function, Frustum154 automatically selects the best subbands for the problem.

Main contributions:

- sEMG signal classification is an important signal processing topic for machine learning; deep learning models have been widely used to classify sEMG signals, achieving excellent accuracy. However, deep models are highly complex. Frustum154 is a hand-crafted, feature-based learning method which can choose the most appropriate model for signal classification problems.
- In order to demonstrate the universal classification ability of the suggested model, three sEMG signal datasets were used; the proposed model achieved over 94% classification for these datasets.

## 2. Material

### 2.1. Material

**Figure 1:**
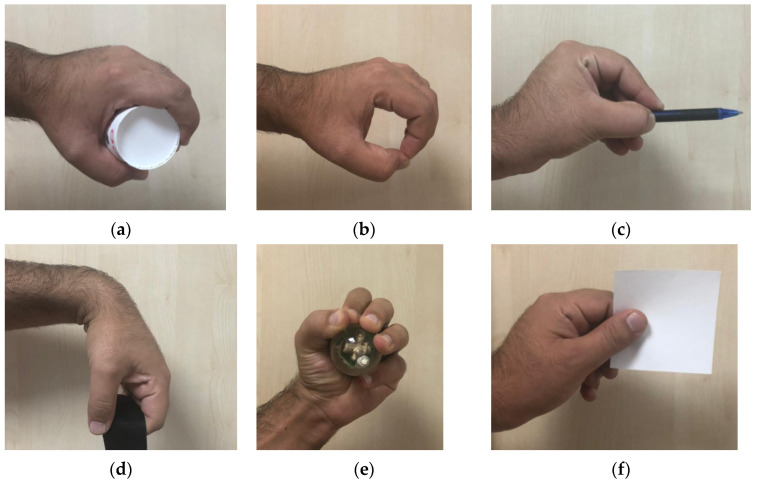
Definition of the proposed Prismatoid pattern. (**a**) Cylindrical grasp-Keeping cylindrical objects. (**b**) Tip-Keeping small objects. (**c**) Palmar-Grasping with palm. (**d**) Hook-Supproting a heavy load. (**e**) Spherical-Keeping spherical objects. (**f**) Lateral-Keeping thin, flat objects.

In this study, an sEMG for basic hand movement dataset from the UC Irvine Machine Learning Repository was used. This dataset contains two subdatasets, from which a third dataset was obtained by fusing the two. More details of these databases are given below. The main purposed of this dataset is to detect six basic hand-movements; (a) Cylindrical (C), (b) Tip (T), (c) Palmar (P), (d) Hook (H), (e) Spherical (S), (f) Lateral (L). Images of these gestures are presented in Figure 1 [38,39].

#### 2.1.1. First sEMG Dataset

The first dataset, named DB1, consisted of data from three healthy female and two healthy male subjects. The ages of subjects range from 20–22 years. These subjects performed six movements. Each subject was asked to perform each of movement for 6 seconds, and movements were repeated 30 times. Thus, 180 pieces of six-seconds, two-channel EMG signals were recorded. This dataset included a total of 900 sEMG signals. The sampling rate of the EMG signal was 500 Hz.

#### 2.1.2. Second sEMG Dataset

The second dataset (DB2) included three days of data from a healthy, male, 22-year-old subject. This subject performed 100 movements for three days. The length of the used sEMG segments was five seconds. This dataset included a total of 1800 sEMG signals. The sampling frequency was 500 Hz, as in the first dataset.

#### 2.1.3. Third sEMG Dataset

DB3 is the fused dataset. It was created by merging DB1 and DB2. It is a homogeneous dataset, with each class containing 450 sEMG signals. Therefore, DB3 contains 2700 sEMG signals in total. In this version, we created a new, large dataset by using both the first and second datasets together.

## 3. Frustum Pattern

Graph-based learning models are very popular in machine learning applications as they can solve difficult problems with a high level of accuracy. Therefore, the effects of such methods should be analyzed. We proposed a hand-modeled learning method using a graph-based feature extractor. This extractor is a graph-based function; the primary objective of this research is to investigate the feature extraction ability of the frustum shape [40] in order to create a new local textural feature generator. The proposed feature extractor was applied to three sEMG datasets to test the feature extraction ability. The main aim of the proposed frustum pattern is to extract hidden patterns from sEMG signals. The vertex and edges of the frustum [40] shape were used to create a new graph, which was utilized as the pattern for the extractor. 

**Figure 2:**
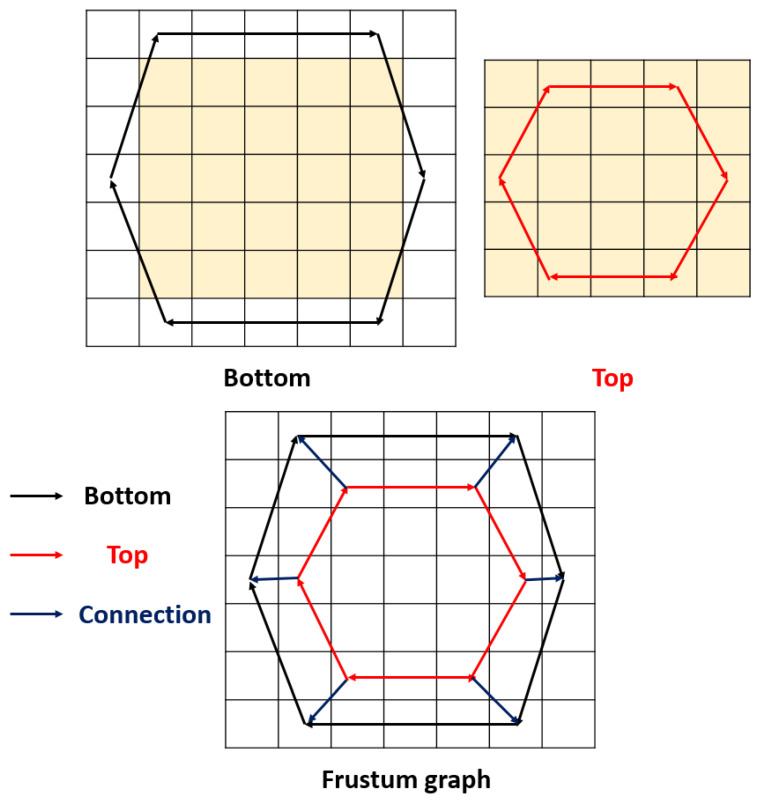
The bottom, top, and frustum graphs used to create the frustum pattern.

This shape was modeled as a feature extraction function. The frustum shape consists of two hexagons. The big hexagon is the bottom of the frustum and the small one is the top. There are six connection edges between the top and the bottom of the hexagons. Therefore, we used two matrices to model this shape as a graph-based pattern. The created bottom and top matrices and the matrices-based patterns are shown in Figure 2.

Figure 2 shows that the proposed frustum pattern uses 7 × 7-sized matrices, as well as two types of edges to generate binary features, i.e., bottom, top, and connection edges. Moreover, the ternary function was utilized as a kernel to generate features. The equations of the ternary bit extractors are given in Equations (1)–(3).

(1) $$t^{1}\left(a,s\right)=\{\begin{array}{c} 0,\;a-s\leq d\\ 1,\;a-s>d \end{array}$$

(2) $$t^{2}\left(a,s\right)=\{\begin{array}{c} 0,\;a-s\geq -d\\ 1,a-s<-d \end{array}$$

(3) $$d=\frac{std\left(S\right)}{2}$$

where $t^{1}\left(.,.\right)$ is the upper ternary function, $t^{2}\left(.,.\right)$ is the lower ternary function, a,s denote input parameters, d is the threshold, std(.) defines standard deviation function and S defines the utilized input signal.

The steps of the proposed frustum pattern are:

***1:*** Divide the signal into overlapping blocks with a length of 49.

(4) obl(j)=S(i+j−1), i∈{1,2,…,Len−48}, j∈{1,2,…,49}

where obl represents the overlapping block and i,j are indices.

***2:*** Create a matrix with a size of 7 × 7 using obl.

(5) mat(k,l)=obl(c), c∈{1,2,…,49}, k∈{1,2,…,7}, l∈{1,2,…,7}

where mat is 7 × 7 sized matrix to apply frustum pattern.

***3:*** Generate bits by applying the frustum pattern and ternary bit extractors.

(6) $$|\begin{array}{c} bit_{k}^{b}\left(1\right)\\ bit_{k}^{b}\left(2\right)\\ bit_{k}^{b}\left(3\right)\\ bit_{k}^{b}\left(4\right)\\ bit_{k}^{b}\left(5\right)\\ bit_{k}^{b}\left(6\right) \end{array}|=t^{k}(\begin{array}{c} mat\left(1,2\right),\;mat\left(1,6\right)\\ mat\left(1,6\right),mat\left(4,7\right)\\ mat\left(4,7\right),mat\left(7,6\right)\\ mat\left(7,6\right),mat\left(7,2\right)\\ mat\left(7,2\right),mat\left(4,1\right)\\ mat\left(4,1\right),mat\left(1,2\right) \end{array}),k\in \left\{1,2\right\}$$

(7) $$\left|\begin{array}{c} bit_{k}^{t}\left(1\right)\\ bit_{k}^{t}\left(2\right)\\ bit_{k}^{t}\left(3\right)\\ bit_{k}^{t}\left(4\right)\\ bit_{k}^{t}\left(5\right)\\ bit_{k}^{t}\left(6\right) \end{array}|=t^{k}(\begin{array}{c} mat\left(2,3\right),\;mat\left(2,5\right)\\ mat\left(2,5\right),mat\left(4,6\right)\\ mat\left(4,6\right),mat\left(6,5\right)\\ mat\left(6,5\right),mat\left(6,3\right)\\ mat\left(6,3\right),mat\left(4,2\right)\\ mat\left(4,2\right),mat\left(2,3\right) \end{array}\right)$$

(8) $$\left|\begin{array}{c} bit_{k}^{c}\left(1\right)\\ bit_{k}^{c}\left(2\right)\\ bit_{k}^{c}\left(3\right)\\ bit_{k}^{c}\left(4\right)\\ bit_{k}^{c}\left(5\right)\\ bit_{k}^{c}\left(6\right) \end{array}|=t^{k}(\begin{array}{c} mat\left(1,2\right),\;mat\left(2,3\right)\\ mat\left(1,6\right),mat\left(2,5\right)\\ mat\left(4,7\right),mat\left(4,6\right)\\ mat\left(7,6\right),mat\left(6,5\right)\\ mat\left(7,2\right),mat\left(6,3\right)\\ mat\left(4,1\right),mat\left(4,2\right) \end{array}\right)$$

where $bit_{k}^{b},\;bit_{k}^{t}$ and $bit_{k}^{c}$ are the *k*^th^ bottom, top, and connection bits.

***4:*** Calculate map signal by transforming bits to decimal numbers.

(9) $$map_{k}^{b}\left(i\right)=\overset{6}{\underset{j=1}{\sum }}bit_{k}^{b}\left(j\right)\times 2^{j-1}$$

(10) $$map_{k}^{t}\left(i\right)=\overset{6}{\underset{j=1}{\sum }}bit_{k}^{t}\left(j\right)\times 2^{j-1}\;\;$$

(11) $$map_{k}^{c}\left(i\right)=\overset{6}{\underset{j=1}{\sum }}bit_{k}^{c}\left(j\right)\times 2^{j-1}$$

***5:*** Extract histograms of the six map signals. Each histogram has 64 elements.

***6:*** Merge the extracted histograms to create a feature vector with a length of 384.

(12) $$fv\left(f+64\times \left(h-1\right)\right)=H^{h}\left(f\right),\;f\in \left\{1,2,\ldots ,64\right\},\;h\in \left\{1,2,\ldots ,6\right\}$$

where fv is the features extracted by using the frustum pattern.

## 4. The Proposed Learning Model: Frustum154

**Figure 3:**
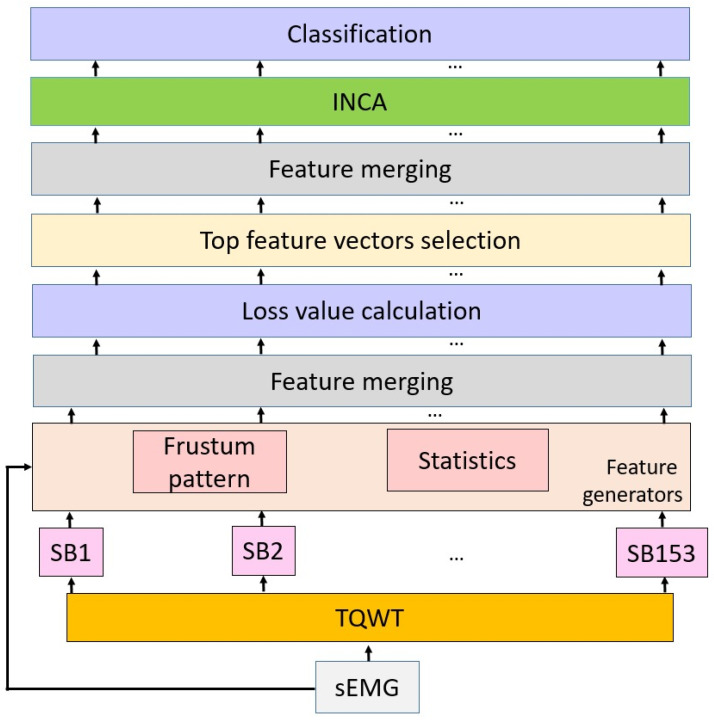
Graphical summary of Frustum154. In the first step, TQWT with multiple parameters is applied to a sEMG signal and 153 subbands (SBs) are calculated. Then, 154 feature vectors (153 subbands + sEMG) are created by applying the proposed frustum pattern and statistical feature extractor. The Frustum pattern generates 384 features, while 30 features are created using statistics. Therefore, the length of each feature vector is computed as 414. By deploying a shallow classifier with 10-fold cross-validation, misclassification rates (loss values) are calculated and the top 20 features are selected according to the loss values. These top features are merged and a feature vector comprising 414 × 20 = 8280 features is obtained, from which INCA chooses the most informative ones, which these are classified using kNN or SVM with 10-fold cross-validation.

The main objective of the proposed model is to achieve excellent classification ability with biomedical signals for classification problems. The model comprises a feed-forward network and a hand-modeled architecture. The used architecture has feature extraction, feature selection, and classification phases. A machine learning model is proposed for feature extraction, comprising a multilevel method. Effective wavelet decomposition (TQWT) is utilized as a decomposition method. By using TQWT, 153 subbands are generated. Two feature extraction functions are used to generate fused features, i.e., a statistical generator and frustum pattern. Using these functions, 154 feature vectors (a raw sEMG signal and 153 subbands) are generated. Misclassification rates of these vectors are calculated using kNN and SVM classifiers (herein, kNN and SVM are utilized as loss functions) and a loss array is created. The top 20 feature vectors are selected using loss values, which are merged to create the final feature vector. INCA is applied to automatically choose the most discriminative features. In the classification phase, kNN or SVM are used to demonstrate the excellent classification ability of the created features. A graphical summary of the proposed model is shown in Figure 3.

Figure 3 describes the proposed model. Pseudocode of the presented model is shown in Algorithm 1, and the transition table of this learning model is given in Table 1.
**Algorithm 1:** Pseudocode of the introduced Frustum154.Input: sEMG datasetOutput: Results.00: Read each sEMG signal from datasets.01: Merge the channels02: Apply for TQWT decomposition to calculate 153 subbands.03: Extract statistical and texture features from sEMG signal and subbands.04: Obtain a 154-feature vector with a length of 414 (=384 + 30). 05: Calculate the misclassification rate of each feature vector.06: Choose the best 20 feature vectors using the calculated misclassification rates.07: Merge the 20 feature vectors selected to create final feature vector. 08: Choose the most informative feature by applying INCA.09: Classify the chosen features using kNN or SVM with 10-fold cross-validation.

### 4.1. Feature Generation

The most complex and first phase of the Frustum154 is feature extraction. In this paper, a machine learning method is proposed as a feature extraction method, since this phase uses feature creation and classification methods together to generate the most appropriate features. The concatenated sEMG signal (channels 1 and 2 were merged to obtain the sEMG signal) and TQWT subbands (153 subbands were generated) were utilized for feature generation. By deploying this feature extractor (Frustum Pattern and Statistics), textural and statistical features were generated from the 154 signals (raw sEMG signal and 153 TQWT subbands). Therefore,154 feature vectors were created, which were then merged to create the final feature vector. To better explain the presented feature generation method, the steps of this process are given in below.

***Step 0:*** Read sEMG signals and concatenate channels to obtain the input sEMG signal.

(13) $$sEMG=conc\left(Ch_{1},Ch_{2}\right)$$

Herein, the concatenated sEMG describes the sEMG signal, $Ch_{1}$ and $Ch_{2}$ are the first and second channels of the sEMG signal and conc(.) is the concatenation function.

***Step 1:*** Decompose sEMG using the TQWT decomposition model. We used multiple parameters TQWT.

(14) SB1=TQWT(sEMG,1,2,6)

(15) SB2=TQWT(sEMG,2,4,24)

(16) SB3=TQWT(sEMG,3,6,46)

(17) SB4=TQWT(sEMG,4,8,73)

(18) SB=[SB1,SB2,SB3,SB4]

where SB1,SB2,SB3,SB4 are generated wavelet coefficients (subbands) by applying TQWT with four variable parameters. By applying these parameters-based TQWT (TQWT(.)), 7, 25, 47, and 74 subbands are created. These subbands are collected in a structure (SB) which contains 153 subbands. This step is parametric. Variable parameters can be used in this phase.

***Step 2:*** Extract features by deploying the proposed Frustum pattern and statistical features.

(19) $$fev^{1}=conc\left(FP\left(sEMG\right),SE\left(sEMG\right)\right)$$

(20) $$fev^{j+1}=conc\left(FP\left(SB^{j}\right),SE\left(SB^{j}\right)\right),\;j\in \left\{1,2,\ldots ,153\right\}\;\;$$

where fev are feature vectors and this model creates 154 feature vectors, FP(.) is frustum pattern and SE(.) is the statistical extractor. We used 15 statistical moments in the used statistical feature extractor; the used moments are tabulated in Table 2.

These statistical moments (see Table 2) were applied to the raw signal and absolute values of the signal.

Thirty statistical features were generated by applying these statistical moments. In this respect, FP(.) generated 384 and SE(.) extracted 30 features from each subband/sEMG signal. By merging these features, 414 (=384 + 30) features were generated from each input (subband/sEMG).

***Step 3:*** Normalize features using min–max normalization.

(21) $$fev^{k}=\frac{fev^{k}-min\left(fev^{k}\right)}{max\left(fev^{k}\right)-min\left(fev^{k}\right)},\;k\in \left\{1,2,\ldots ,154\right\}$$

***Step 4:*** Apply a loss generation function (kNN or SVM with 10-fold cross-validation) to generate features and calculate the loss array. The main objective of this step is to select the most significant subbands for feature extraction. In order to choose the most significant subbands according to the proposed frustum pattern and statistical feature extraction (see Table 2), loss values had to be calculated. Therefore, shallow classifiers were utilized as the loss value generator.

***Step 5:*** Choose the top 20 feature vectors and concatenate them to obtain features with a length of 8280. This architecture is parametric and we selected the top 20 feature vectors to create the final feature vector. A variable number of features or a threshold point can be used to create the final feature vector.

After creating the final feature vector, the optimal number of features was chosen using INCA selector; details are presented in Section 4.2.

### 4.2. Feature Selection

Herein, an iterative feature function, i.e., INCA, was used to select the best feature combination. This is an iterative version of the NCA classifier; its primary purpose is to solve automated optimal feature vector selection using NCA, since NCA cannot select the best number of features without using the trial and error method. INCA was presented by Tuncer et al. [34] in 2020, and is a very effective feature selector. The parameters of the applied INCA are defined in Table 1. For cubic SVM and kNN (1NN with L1-norm) with 10-fold cross-validation were used. For DB1 (first database), kNN is the best classifier. For others (DB2 and DB3), the best loss value generator is Cubic SVM. This generator chooses 413 features and selects the best combination according to misclassification rates. In this work, three datasets were used for testing. INCA chose 279, 277, and 295 features for the DB1, DB2, and DB3 datasets, respectively. The steps for the NCA are given below.

***Step 6:*** Deploy INCA to select the most informative features.

### 4.3. Classification

kNN and SVM classifiers were used and results were obtained. Therefore, the most appropriate classifier was selected to solve the problem. We used two classifiers and the proposed Frustum154 chose the most effective one. The properties of the used classifiers are tabulated in Table 1.

***Step 7:*** We then calculated the results using the kNN or SVM classifier. The hyperparameters of the used classifiers are as follows. For the kNN classifiers, k is 1, distance is Manhattan and voting is none. The hyperparameters of the SVM classifier are as follows: Kernel scale is auto, kernel is 3rd degree polynomial, C (box constraint) value is 1 and coding is one-vs-one. Moreover, ten-fold CV was used to validate these classifiers.

## 5. Experimental Protocol

### 5.1. Experimental Setup

In this paper, three publicly-available sEMG signal datasets were used to evaluate Frustum a pattern-based classification model. The presented model is self-organized. To implement it, MATLAB 2021b was used. We programmed this model using the following functions: main, TQWT, Frustum_Pattern, statistics, loss_calculator, feature_vector_selector, INCA and classification. This model was implemented on a personal computer (PC) with a simple configuration, as it is lightweight and there is no need to use any unusual hardware.

### 5.2. Validation

The presented model is a classification model. In the classification and loss value generation phases, 10-fold cross-validation was used to obtain robust results. In this validation technique (10-fold cross-validation), the observations were divided randomly into 10 folds, and the average value of the results was calculated.

### 5.3. Results

To evaluate the presented Frustum154, three sEMG signals datasets were used for the general classification results. We used recall, precision, F1-score, and accuracy performance metrics to obtain measurements. The present model can select the best classifier according to the problem. The results of the DB1 were calculated by utilizing the kNN classifier. SVM was used for the other two datasets (DB2 and DB3). Furthermore, 10-fold cross-validation was utilized as a validation model to obtain robust classification results. The calculated confusion matrices are tabulated in Table 3.

Table 3 shows that the proposed Frustum154 achieved 100% class-wise accuracies (recall) for cylindrical and spherical movements, as well as 100% F1-score for spherical movement. The confusion matrix for DB2 is tabulated in Table 4.

For DB2, the best class was spherical movement (S), for which Frustum154 achieved 99.67% recall. The worst categories were Palmar and Lateral, which reached 91% classification accuracies.

The last signal dataset was DB3; this is a merged dataset. Table 5 shows the confusion matrix for DB3.

Spherical movement was the best class for DB3, as was the case with DB1 and DB2. The worst category was Tip, with Frustum154 achieving 91.11% recall.

Based on Table 3, Table 4 and Table 5, the overall results are tabulated in Table 6.

Table 6 shows that Frustum154 achieved 98.89%, 94.94% and 95.30% classification accuracies for DB1, DB2 and DB3, respectively.

**Figure 4:**
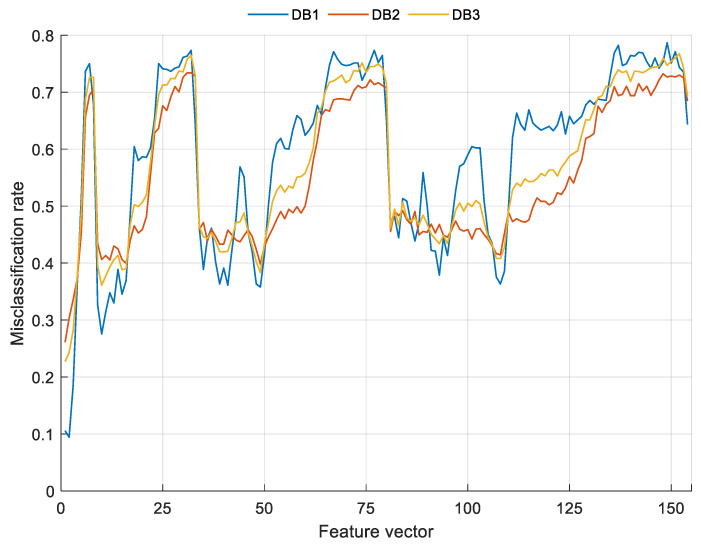
The calculated misclassification rates.

Moreover, the differences among the used feature selection methods (we used two feature selection approximations, i.e., loss value-based selection in the feature extraction and the INCA model) are explained below. To choose the most appropriate feature vectors, loss values were calculated; these error rates are shown in Figure 4.

**Figure 5:**
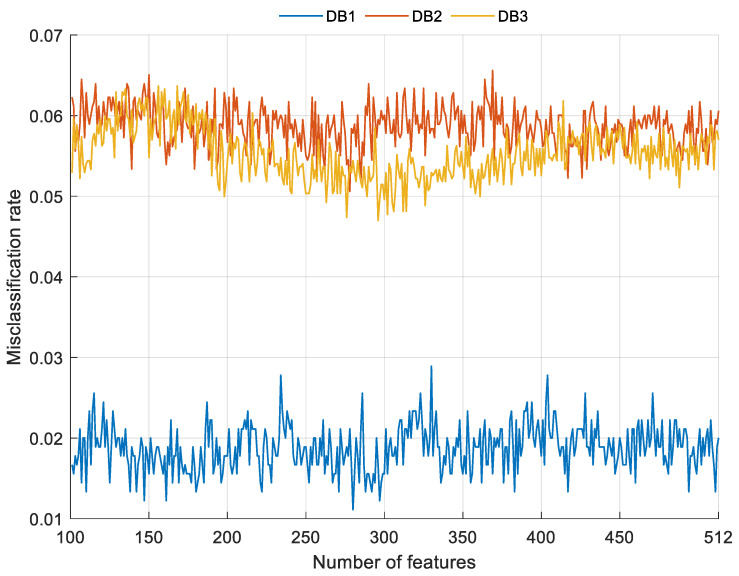
INCA feature selection process. Misclassification rates according to the number of features.

As shown in Figure 4, the best accuracies of the individual feature vector for DB1, DB2, and DB3 were calculated as 90.56%, 73.89%, and 77.30% respectively. Feature merging (top 20 features concatenation) and INCA were applied to increase these classification accuracies. The INCA feature selection process is shown in Figure 5.

The feature merging and INCA processes increased the accuracy rates from 90.56%, 73.89%, and 77.30% to 98.89%, 94.94%, and 95.30% for DB1, DB2, and DB3 respectively.

### 5.4. Time Complexity Analysis

A time complexity analysis was conducted. Big theta notation was used to present the general results. By using this notation, the training and testing complexities of our presented Frustum154 could be computed; see Table 7.

Here, variables were used to calculate asymptotic notation as follows. t is the number of subbands, n defines the length of the sEMG signal, d is the number of observations, k defines the time complexity coefficient of the parameter, f is the number of features and m is the number of iterations in INCA. As shown in Table 7, the time complexity of this model is linear.

## 6. Discussion

This research presents a new classification network to detect six basic hand movements using sEMG signals. The proposed model, Frustum154, creates 154 feature vectors and selects the 20 most appropriate feature ones to create final features. The results (see Section 5) clearly demonstrate the success of the presented feature generation network and a new attribute, i.e., shoelaces, was investigated. To better illustrate the success of the model, a performance comparison was performed; the results are tabulated in Table 8.

To the best of our knowledge, to date, no method has utilized DB2 and DB3. Therefore, we cannot perform any comparisons of such cases. We obtained over 94% classification accuracies for all datasets, indicating the high classification rates of our model. Comparisons were made using DB1, since this is the simplest dataset. Subasi and Qaisar [41] presented a statistical feature extraction-based model using DB1 which achieved 94.11% accuracy. They showed the classification ability of the statistical features but their classification result was not good. Nishad et al. [17] presented a TQWT and statistical feature extraction-based model. They used DB1 to calculate the results. DB1 contains five subjects, and the authors calculated the results for each. To calculate the overall performance, they used the average values. They did not use all of the dataset to get general results; other models [42,43] have used the same strategy. Coskun et al. [19] presented a one-dimensional, CNN-based deep learning model and achieved 94.94% accuracy. Tsinganos et al. [44] introduced a CNN-based deep learning model but did not achieve satisfactory classification results. Deep learning models have high computational complexity, since many parameters need to be optimized. A self-organized hybrid, hand-crafted feature-based model is presented in this research. To show the classification ability of the presented Frustum pattern-based model, three sEMG datasets for basic hand-movements were used; our model yielded excellent classification results. It is worth noting that:

- The feature generation capabilities of the frustum pattern/graph were investigated and the sEMG classification was found to be highly successful;
- To maximize the effectiveness of TQWT, multiple parameter-based TQWT was used and 153 subbands were generated;
- An improved feature selector (INCA) was used;
- A novel hand-modeled learning method is proposed;
- The proposed Frustum154 achieved a higher classification rate than deep learning models (see Table 8);
- The present model showed good general classification success.

The proposed model could also be applied to more complex and larger datasets; this will be explored in future work.

## 7. Conclusions

The objective of a machine learning model is to achieve excellent classification with low execution time; however, there is usually a tradeoff. To overcome this problem, a self-organized model has been presented and three sEMG signal datasets have been used to depict the general efficacy of the presented model. A novel, hand-modeled feature selection-based, basic hand-movement classification network using a multilevel feature generation method is presented. This approach was inspired by deep feature networks and it has low- and high-level feature generation capabilities. The proposed Frustum154 method generates 154 features, selects the top 20 feature vectors, and chooses the most discriminative ones by deploying the INCA selector. FrustumNet154 has been tested on three datasets, achieving good performance with all three. The accuracies for these datasets were 98.89%, 94.94% and 95.30%, respectively. In the literature (see Table 8), the first sEMG dataset was used to calculate the classification results. This model solved this problem and all of them were used to test the performance of Frustum154. Most prior models calculate results for each subject and then use the average to achieve good classification ability. In contrast, Frustum154 calculates the results using all of the sEMG observations, allowing it to achieve superior classification rates. This research used two channeled sEMG signals. Therefore, this model could be employed with low-cost exoskeleton prosthetic hands (EPH) or smart gloves. Based on our findings, the proposed approach is a successful classification model for one-dimensional signals (sEMGs). Our approach motivates new low-cost and smart EPHs and smart gloves which could be used in physiotherapy and orthopedics clinics. New smart sEMG signal monitoring applications can be derived by applying our presented model. Other one-dimensional signals can also be classified by applying this model.

## Figures and Tables

**Table 1:** Transition table of the presented Frustum154.

Operation	Parameter	Output
Channel merging	Two channels	The used datasets comprise two-channeled sEMG signals. These channels are concatenated to use both channels.
TQWT	Q = 1, 2, 3, 4 r = 2, 4, 6, 8 J = 6, 24, 46, 73	153 subbands
Frustum pattern	Forty-nine overlapping blocks are used. The kernel function is ternary and the threshold value is chosen as half of the standard deviation of the signal	154 feature vectors with a length of 384
Statistical feature extraction	We applied well-known statistical moments	154 feature vectors with a length of 30
Feature merging		154 feature vectors with a length of 414
Normalization	Min-max normalization	154 feature vectors are normalized
Loss value generation	Cubic SVM and kNN (1NN with L1-norm) with 10-fold cross-validation. Herein, greedy model has been used. For DB1 (first database), kNN is the best classifier. For others (DB2 and DB3), the best loss value generator is Cubic SVM.	154 loss values
Top 20 feature vectors selection	Loss array	20 feature vectors
Feature merging	Concatenation function	Final feature vector with a length of 8280
INCA selector	Iteration range: [100, 512] Classifier: SVM or kNN (greedy search-based)	Length of the chosen feature vectors DB1: 279 DB2: 277 DB3: 295
Classification	kNN: k is 1, distance is Manhattan and voting is none. Validation is 10-fold CV. SVM: Kernel scale is auto, kernel is 3rd degree polynomial, C value is 1 and coding is one-vs-one. Validation is 10-fold CV.	Predicted values

More explanations of the FrustumNet41 are given in subsections.

**Table 2:** The used statistics for feature extraction.

No.	Statistics	No.	Statistics	No.	Statistics
1	Mean	6	Maximum	11	Kurtosis
2	Median	7	Minimum	12	Skewness
3	Variance	8	Standard deviation	13	Higuchi
4	Shannon entropy	9	Range	14	Energy
5	Log entropy	10	Sure entropy	15	Root mean square error

**Table 3:** The confusion matrix of the DB1.

True Label	Predicted Label					
	C	H	L	P	S	T
C	150	0	0	0	0	0
H	0	149	0	1	0	0
L	0	0	147	1	0	2
P	0	0	1	149	0	0
S	0	0	0	0	150	0
T	1	1	1	2	0	145
Recall (%)	100	99.33	98	99.33	100	96.67
Precision (%)	99.34	99.33	98.66	97.39	100	98.64
F1 (%)	99.67	99.33	98.33	98.35	100	97.64

**Table 4:** The confusion matrix of the DB2.

True Label	Predicted Label					
	C	H	L	P	S	T
C	297	1	0	0	2	0
H	3	291	0	2	0	4
L	1	1	273	18	0	7
P	0	2	19	273	0	6
S	1	0	0	0	299	0
T	0	1	11	12	0	276
Recall (%)	99	97	91	91	99.67	92
Precision (%)	98.34	98.31	90.10	89.51	99.34	94.20
F1 (%)	98.67	97.65	90.55	90.25	99.50	93.09

**Table 5:** The confusion matrix of the DB3.

True Label	Predicted Label					
	C	H	L	P	S	T
C	442	5	0	0	3	0
H	4	431	0	7	2	6
L	0	0	421	19	0	10
P	0	2	14	425	0	9
S	5	1	0	0	444	0
T	2	6	18	14	0	410
Recall (%)	98.22	95.78	93.56	94.44	98.67	91.11
Precision (%)	97.57	96.85	92.94	91.40	98.89	94.25
F1 (%)	97.90	96.31	93.24	92.90	98.78	92.66

**Table 6:** Overall results (%) of the proposed Frustum154 for the used datasets.

Performance Metrics	DB1	DB2	DB3
Accuracy	98.89	94.94	95.30
Precision	98.89	94.97	95.32
F1	98.89	94.95	95.30

**Table 7:** Time complexity computation of the proposed Frustum154.

Phase	Step	Training	Test
Feature extraction	Feature vectors creation using TQWT and frustum pattern	θ(tndlognd)	θ(tnlogn)
	Feature vector selection using loss values	θ(tdfk)	θ(1)
	Feature concatenation	θ(fd)	θ(1)
Feature selection	INCA	θ(fdkm)	θ(h)
Classification	kNN/SVM	θ(fdk)	θ(k)
Total		θ(tndlognd+tdfk+fdkm)	θ(tnlogn+h+k)

**Table 8:** Results (%) of prior sEMG signal classification methods and those of Frustum154.

Study	Method	Dataset	Accuracy (%)
Subasi and Qaisar [41]	Statistical feature extraction	DB1	94.11
Nishad et al. [17]	Statistical (entropy) feature extraction with TQWT decomposition	DB1	98.55
Iqbal et al. [42]	Singular value decomposition and principal component analysis (SVD+PCA) and kNN classifier	DB1	86.71
Sapsanis et al. [43]	Statistics and emprical mode decomposition (EMD) transformation	DB1	86.64
Coskun et al. [19]	One dimensional convulotional neural network (1D-CNN)	DB1	94.94
Tsinganos et al. [44]	Convolutional neural network	DB1	72.06
Rabin et al. [16] method	Short time Fourier transform-based feature generation and principle component analysis/diffusion map-based feature reduction + kNN	DB1	76.4
Frustum154		DB1	98.89
		DB2	94.94
		DB3	95.30

## Data Availability

The used dataset was downloaded from https://archive.ics.uci.edu/ml/datasets/sEMG+for+Basic+Hand+movements, accessed on 20 January 2022. We thank UCI Machine Learning Repository.
